# Identification of 99% of *CFTR* gene mutations in Bulgarian‐, Bulgarian Turk‐, and Roma cystic fibrosis patients

**DOI:** 10.1002/mgg3.696

**Published:** 2019-06-27

**Authors:** Guergana Petrova, Nadezhda Yaneva, Jana Hrbková, Malgorzata Libik, Alexey Savov, Milan Macek

**Affiliations:** ^1^ Pediatric Clinic, University Hospital Alexandrovska Sofia Bulgaria; ^2^ Pediatric Department Medical University Sofia Sofia Bulgaria; ^3^ University Specialized Hospital for Active Treatment in Obstetrics and Gynecology, 'Maichin dom', National Genetic Laboratory Sofia Bulgaria; ^4^ Medical University Sofia Sofia Bulgaria; ^5^ Department of Biology and Medical Genetics University Hospital Motol Prague Czech Republic; ^6^ Faculty of Medicine of Charles University Prague Czech Republic

**Keywords:** Bulgaria, Bulgarians, Bulgarian Turks, cystic fibrosis, *CFTR* gene, Roma

## Abstract

**Background:**

The spectrum and frequencies of CFTR mutations causing Cystic fibrosis (CF) varies among different populations in Europe, and beyond.

**Methods:**

We identified 98.9% of all *CFTR* mutations in a representative cohort of 140 CF patients comprising 107 Bulgarian‐ (BG), 17 BG Turk‐, and 16 BG Roma cases. The compiled clinical and genotype dataset includes 110 previously analyzed patients with 30 cases currently analyzed for rare *CFTR* variants by massively parallel sequencing of the entire *CFTR* coding region and adjacent introns combined with the analysis of intra‐*CFTR* rearrangements.

**Results:**

Altogether 53 different mutations, of which 15 newly identified in the BG CF population, were observed. Comparison of clinical and laboratory data between individual BG ethnic groups proved that BG Roma have a more severe nutritional status and are younger than other CF patients, as well as that the spectrum mutations differs between them.

**Conclusion:**

This collaborative study improves genetic counselling in BG, facilitates introduction of multitier CF neonatal screening and fosters public health measures for improvement of care in the Roma CF population.

## 1 INTRODUCTION

Cystic fibrosis (CF; MIM# 219700) is an autosomal recessive rare disease caused by CF‐causing variants (henceforward mutations) in the cystic fibrosis transmembrane conductance regulator gene (*CFTR;* MIM#602421). The spectrum and frequencies of mutations varies among different populations in Europe, and beyond (Bobadilla, Macek, Fine, & Farrell, 2002; Orenti et al., 2018).

The at birth prevalence of cystic fibrosis (CF) in Bulgaria (BG) was estimated using epidemiological methods as being 1:3,600 live births (Savov, 2011). In 2017 this estimate was substantiated by 20 newly clinically diagnosed cases in a total of 64,359 live births (data from the national BG CF registry/BGCFR/; this study). Nevertheless, the updated at birth prevalence of CF is likely not accurate since BG has so far not implemented a nationwide cystic fibrosis neonatal screening program (CFNBS) (National Centre of Public Health & Analyses, 2014).

Population genetic studies provided evidence that BG share about in about 45% of their genetic variation with the Balto‐Slavic populations. In addition, the second half of the BG “genetic legacy” is of Mediterranean origin with minor influences from the Caucasus‐, M. East‐, and N. Africa (Hellenthal et al., 2014). Furthermore, in the history BG underwent multiple immigration waves mainly from current Turkey (TK) and Greek Thrace. The last nationwide census (2011) reports three major self‐reported ethnic groups comprising BG (85%), Bulgarian Turks (BGTK; 8.8%), and Bulgarian Roma (BGRM; 4.9%) within country of 7 million inhabitants (National Statistical Institute of the Republic of Bulgaria, 2011).

As of December 2018, 201 CF patients were reported in the BGCFR, whereas more than half of them are regularly followed up at the University Hospital Alexandrovska (Sofia). This University centre takes care of about two thirds of all known cases in BG and runs the BGCFR.

Thirty cases where one or both *CFTR* gene mutations *in trans* remained unidentified following the initial screening for common population specific *CFTR* mutations carried out by collaborating National Genetic Laboratory in Sofia (Angelicheva et al., 1997; Savov, 2011; Savov et al., 1995) were examined in collaboration with the Department of Biology and Medical Genetics (Prague, Czech Republic; CZ). There the complete analysis of the *CFTR* gene coding region, including analysis of intra‐*CFTR* rearrangements and of adjacent intronic sequences, was performed according to an established methodology (Křenková et al., 2013).

The aim of this study was to report distribution of CF‐causing mutations in a representative group of BG CF patients, divided according to their ethnicity and thus representing constitutive BG‐, BGTK‐, and BGRM populations.

This study supersedes previously published limited reports (Angelicheva et al., 1997; Savov, 2011; Savov et al., 1995), both in terms of the nationwide representativeness, overall number of patients examined and comprehensiveness of *CFTR* gene molecular genetic analysis by massively parallel sequencing (MPS) complemented by intra‐*CFTR* rearrangement analysis. We have also carried out genotype phenotype correlations stratified by individual BG subpopulations.

## 2 METHODS

**Figure 1:**
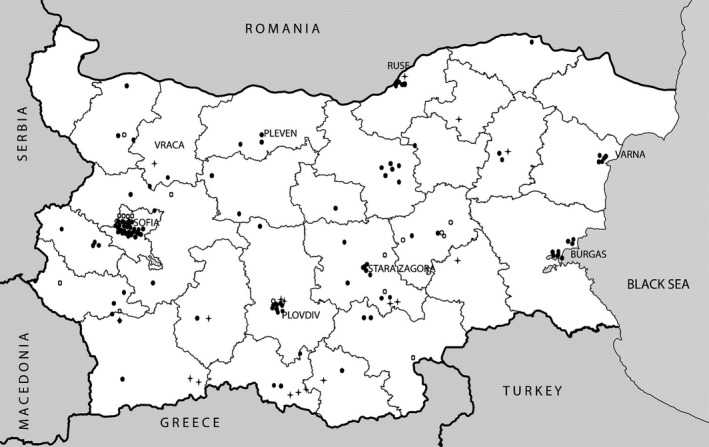
Regional origin of examined Bulgarian‐, Bulgarian Turk‐, and Bulgarian Roma CF patients. Legend: Regional CF patient distribution (BG ●, BGTK + and BGRM □) is based on postal codes of their domicile. Respective population density in BG according to Eurostat data (ec.europa.eu/eurostat and www.nsi.bg/sites/default/files/files/data/table/BG_grid_POP_1K_2011_poster_0.pdf; Accessed January 12, 2019)

The clinical diagnosis of CF was established in 140 unrelated patients, comprising three major BG ethnic groups (BG‐107, BGTK‐17, and BGRM‐16 cases), according to clinical and laboratory consensus diagnostic criteria (Farrell et al., 2008). An outline of their key demographic, clinical, and laboratory characteristics according to BGCFR data (2017) is presented in Table 1, and their geographic origin is shown on Figure 1. Initially, all cases were examined for the most common “European” CF mutations using “in‐house” methods and Sanger DNA sequencing of selected *CFTR* exons with this combined approach leading to the identification of both *CFTR* mutations in approx. 80% of all cases as published reported (Angelicheva et al., 1997; Savov, 2011; Savov et al., 1995). GenBank reference sequence and version number for the gene studied was NM_00492.3 (CFTR).

**Table 1:** Overview of demographic, clinical, and laboratory characteristics of Bulgarian‐, Bulgarian Turk‐, and Bulgarian Roma CF populations

	Bulgarian	Bulgarian Turks	Roma	P^a^	Roma (homozygous for mutation F508del)	Bulgarian (homozygous for mutation F508del)	P^b^
Number of patients	107	17	16		14	21	
Male/female	60:47	9:8	9:7	0.99	7:7	10:11	0.89
Mean age (years); ±*SD* (min–max)	18.25 ± 12.35 (0.56–62.68)	15.36 ± 11.05 (2.08–29)	**9.51 ± 6.2** **(0.88–19.6)**	**0.041**	**9.53 ± 5.74** **(1.48–19.6)**	20.45 ± 10.56 (5.32–41.76)	**0.014**
Mean age of diagnosis (years); ±*SD*	4.73 ± 8.44	5.77 ± 10.31	**1.71 ± 2.76**	**0.02**	1.9 ± 2.91	2.26 ± 3.23	0.36
Number of patients older than 18 years (%)	54 (50.46%)	6 (35.29%)	2 (12.5%)	0.135	2 (14.28%)	11 (52.38%)	0.10
Patients with 2 *CFTR* alleles in *trans*: 2/1/0	104/3/0	17/0/0	16/0/0	0.99	14/0/0	21/0/0	n.a.
Homozygous for mutation F508del %	19.62%	23.53%	**87.5%**	**0.001**	100%	100%	n.a.
Compound heterozygous mutation F508del %	53.27%	47.05%	6.25%^d^	*0.054*	n.a.	n.a.	n.a.
Non‐F508del patients %	27.11%	29.42%	6.25%^e^	0.34	n.a.	n.a.	n.a.
Mean BMI Z score; ±*SD*	−1.29 ± 1.46	−0.11 ± 1.22	**−1.83 ± 1.52**	**0.004**	−1.25 ± 1.28	−1.75 ± 1.56	0.24
Mean FEV_1_ (% predicted); ±*SD*	65.85 ± 28.63	74.24 ± 18.73	73.2 ± 28.36	0.05	78.25 ± 25.63	55.3 ± 26.28	0.51
Chronic *P. aeruginosa* lung colonization [yes:no] (%)	63:42 (60%)	10:7 (58.82%)	7:9 (43.75%)	0.53	6:8 (42.85%)	17:4 (80.95%)	0.27
CF liver disease [yes:no] (%)	20:87 (18.69%)	1:16 (5.88%)	3:13 (18.75%)	0.40	3:11 (21.42%)	6:15 (28.51%)	0.71
CF‐related diabetes mellitus [yes:no] (%)	5:102 (4.67%)	0:17 (0%)	1:16 (6.25%)	0.05	1:13 (7.14%)	1:20 (4.76%)	0.79
Pancreatic sufficient cases [yes:no]	2:105	0:17	0:16	0.62	0:14	0:21	n.a.
Female patients that gave birth (%)	7 (14.89%)	2 (25%)	0 (0%)	0.23	0 (0%)	0 (0%)	n.a.
Patients born with meconium ileus (%)	8 (7.47%)	1 (5.88%)	1 (6.25%)	0.92	0 (0%)	1 (4.76%)	0.92
Concomitant non CF‐related diseases [yes:no] (%)^c^	5:102 (4.67%)	4:13 (23.52%)	1:15 (6.25%)	0.083	1:13 (6.66%)	1:20 (4.76%)	0.79
Patients after lung transplantation	2	0	0	n.s.	0	0	n.a.

Note

Presented data are drawn from the BGCFR 2017 datasets.

Statistically significant differences highlighted in bold text. %: percentage rounded up to max. 2 digits after the full stop (thus may not add up exactly to 100%).

BMI, body mass index; FEV_1_, forced expiratory volume for 1 s; n.a., not applicable; n.s., not statistically significant; *SD*, standard deviation.

a Comparison between the three groups of patients (Bulgarian‐, Bulgarian Turk‐, and Bulgarian Roma CF patients); Statistical methods used were nonparametric tests (Kruskal–Wallis and Chi‐square tests).

b Comparison between the two groups of homozygous patients (Bulgarian vs. Bulgarian Roma); Statistical methods used: Mann–Whitney and Chi‐square tests; Concomitant non‐CF related diseases.

c Intellectual disability (2 cases), epilepsy (2), glomerulonephritis (1), supraventricular tachycardia (1), dilated cardiomyopathy (2), hydrocephalus (1) and brain aneurysm (1).

d The patient's mother had a Bulgarian ancestor, but she self identifies as being of Roma origin.

e The patient's parents had a Turkish ancestor, but both self‐identified as being of Roma origin.

In this study, the 30 patients drawn from the three BG ethnic groups, where one or both CF alleles remained unidentified, were subjected a “cascade” mutation screening approach. First use the panel of the 50 most common *CFTR* variants in the European‐derived populations Elucigene CF‐EU ver.2Tm (Elucigene, UK), followed by MPS‐based analysis of the entire *CFTR* coding region, adjacent splice site junctions, and several introns using a locus‐specific library preparation assay (CFTR NGS assay^™^; Devyser, Sweden; www.devyser.com). MPS sequencing was performed on the MiSeq System^™^ (Illumina, USA; www.Illumina.com). Bioinformatic analysis was carried out using the SOPHiA Platform for Hereditary Disorders^™^ online software (www.sophiagenetics.com). Where applicable positive cases were confirmed by targeted Sanger DNA sequencing on ABI 3130xl DNA Analyser^™^ (ThermoFisher, USA; www.thermofisher.com). Multiplex ligation‐dependent probe amplificiation (MLPA) analysis of intra‐*CFTR* rearrangements and copy number variation was performed by the SALSA MLPA P091 CFTR Assay^™^ followed by analysis of raw data on the proprietary software Coffalyser.Net^™^ (MRC‐Holland, The Netherlands; www.MRC-holland.com). The linkage phase of detected mutations was established by testing less common mutations or suspected complex *CFTR* alleles in index case's parents (data not shown). Variant pathogenicity was assessed according to the CFTR2 database (www.cftr2.org), whereas detected BG mutations were submitted to it in return where applicable. This study was approved by the respective ethics committees of collaborating CZ and BG academic institutions and BG CF patients consented to *CFTR* genotyping.

## 3 RESULTS

Figure 1 visually supports the representativeness of the studied cohort and that there is no regional bias. The number of patients from individual BG regions corresponds to relative population density and respective census data of their domicile. All CF mutations are associated with the classical form of the disease (Table 1; additional detailed clinical and laboratory data are available upon request). In terms of key clinical characteristics of CF patients of BG‐ versus BGTK origin were not significantly different (Table 1). However, BGRM have a more severe nutritional status and are overall of younger age. This significant difference is also apparent when BG versus BGRM F508del homozygous patients are compared (Table 1).

Table 2 depicts genotyping data from a total of 140 BG CF patients of BG‐, BGTK‐, and BGRM origin drawn from the previous reports (*n* = 110) (Makukh et al., 2010; Orenti et al., 2018; Radivojevic et al., 2004) with those generated in this study (*n* = 30; formatted in *italics*). We detected a total of 53 different CFTR variants located throughout all *CFTR* exons, with only 17 being present at a frequency of over 1%. Approximately half of all variants observed (*n* = 28) were private since they were detected only within a single family. Three novel mutations were detected according to the data from the CF Mutation Database (www.genet.sickkids.on.ca/app; Accessed January 12, 2019). From all tested cases with the classical form of the disease only 3 alleles remained unknown (1.07%; Table 2). The population spectra of mutations in the three BG constitutive patient cohorts are presented in Table 2.

**Table 2:** Distribution of *CFTR* variants detected in 107 Bulgarian‐, 17 Bulgarian Turk‐, and 16 Bulgarian Roma CF patients

Standard and colloquial nomenclature for CFTR variants NM_000492.3	BG *N* (%)	BGTK *N* (%)	BGRM *N* (%)	TOTAL *N* (%)
c.1521_1523delCTTp.(Phe508del) **F508del**	110 (51.40)	15 (44.17)	29 (90.62)	154 (55.00)
c.3909C>G p.(Asn1303Lys) **N1303K**	12 (5.60)	—	—	12 (4.29)
c.1624G>T p.(Gly542*) **G542X**	9 (4.21)	1 (2.94)	1 (3.12)	11 (3.93)
c.2052_2053insA p.(Gln685Thrfs*4) **2184insA**	7 (3.27)	1 (2.94)	—	8 (2.89)
c.1040G>C p.(Arg347Pro) **R347P**	1 (0.47)	4 (11.76)	—	5 (1.79)
c.2657+5G>A **2789**+**5G>A**	4 (1.87)	1 (2.94)	—	5 (1.79)
c.3718−2477C>T **3849**+**10kbC>T**	4 (1.87)	—	—	4 (1.43)
c.489+1G>T **621**+**1G>T**	4 (1.87)	—	—	4 (1.43)
c.658C>T p.(Gln220X) **Q220X**	3 (1.40)	1 (2.94)	—	4 (1.43)
c.3846G>A p.(Trp1282X) **W1282X**	3 (1.40)	1 (2.94)	—	4 (1.43)
c.828C>A p.(Cys276*) **C276X**	4 (1.87)	—	—	4 (1.43)
*c.3889dupT* p.(Ser1297Phefs*5) ***4016insT***	—	3 (8.82)	—	3 (1.07)
CFTRdele 18–20	3 (1.40)	—	—	3 (1.07)
c.1545_1546delTA p.(Tyr515*) **1677delTA**	3 (1.40)	—	—	3 (1.07)
c.2051_2052delAAinsG p.Lys684Serfs*38 **2183delAA**>**G**	3 (1.40)	—	—	3 (1.07)
c.1712C>T p.(Leu571Ser) **L571S**	1 (0.47)	2 (5.88)	—	3 (1.07)
*c.532G*>*A* p.Gly178Arg ***G178R***	3 (1.40)	—	—	3 (1.07)
c.174_177delTAGAp.(Asp58Glufs*32) **306delTAGA**	2 (0.93)	—	—	2 (0.71)
*c.1135G*>*T* p.(Glu379*) ***E379X***	2 (0.93)	—	—	2 (0.71)
c.2491G>T p.(Glu831) **E831X**	—	2 (5.88)	—	2 (0.71)
c.3731G>A p.(Gly1244Glu) **G1244E**	2 (0.93)	—	—	2 (0.71)
c.3209G>A p.(Arg1070Gln) **R1070Q**	2 (0.93)	—	—	2 (0.71)
c.1000C>T p.(Arg334Trp) **R334W**	2 (0.93)	—	—	2 (0.71)
*c.1366G*>*T* p.(Val456Phe) ***V456F***	2 (0.93)	—	—	2 (0.71)
**^$^** c.3731G>Tp.(Gly1244Val) **G1244V**+c.2735C>T p.(Ser912Leu) **S912L**	2 (0.93)	—	—	2 (0.71)
*c.54−5940_273*+*10250del21kb* p.(Ser18Argfs*16)*CFTRdele−2.3(21kb)*	2 (0.93)			2 (0.71)
*c.38C*>*T* p.(Ser13Phe*)* ***S13F***	1 (0.47)	—	—	1 (0.36)
p.E54* (c.160G>T)	1 (0.47)	—	—	1 (0.36)
c.254G>A p.Gly85Glu **G85E**	1 (0.47)	—	—	1 (0.36)
CFTRdele 4–11	1 (0.47)	—	—	1 (0.36)
*c.274G*>*A* p.(Glu92Lys) ***E92K***	1 (0.47)	—	—	1 (0.36)
c.442delA p.(Ile148Leufs*5) **574delA**	1 (0.47)	—	—	1 (0.36)
c.579+3A>G **711**+**3A>G**	1 (0.47)	—	—	1 (0.36)
c.650 A>G p.(Glu217Gly) **E217G**	—	1 (2.94)	—	1 (0.36)
c.869+5G>A	1 (0.47)	—	—	1 (0.36)
*c.1202G*>*A* p.Trp401* ***W401X***	—	—	—	1 (0.36)
*c.1393−1G*>*A* ***1525−1G***>***A***	1 (0.47%)	—	1 (3.12)	1 (0.36)
***R1070Q‐S466X*** * c.1397C*>*G*p.(Ser466X)^a^	—		—	1 (0.36)
c.1478A>Gp.(Gln493Arg)**Q493R**	1 (0.47)	—	1 (3.12)	1 (0.36)
*c.1766*+*1G>C* ***1898***+***1 G***>***T***	—	1 (2.94)	—	1 (0.36)
c.1766+3A>G **1898**+**3A>G**	1 (0.47%)	—	—	1 (0.36)
c.1716_1719 delCTCT+c.1714G>A(p.Asp572Asn)^a^	1 (0.47%)	—	—	1 (0.36)
c.2052delA p.Lys684Asnfs*38 **2184delA**	1 (0.47%)	—	—	1 (0.36)
*c.3160C*>*G* p.(His1054Asp) ***H1054D***	—	1 (2.94)	—	1 (0.36)
(c.3205G>A)p.(Gly1069Arg)**G1069R** *c.3454G*>*C* p.(Asp1152His) ***D1152H***	1 (0.47%)	—	—	1 (0.36)
c.3472C>T p.(Arg1158*) **R1158X**	1 (0.47%)	—	—	1 (0.36)
c.3484C>T p.(Arg1162*) **R1162X**	1 (0.47%)	—	—	1 (0.36)
c.3584A>C p.(Asn1195Thr) **N1195T**	1 (0.47%)	—	—	1 (0.36)
*c.4004T*>*C* p.Leu1335Pro ***L1335P***	1 (0.47%)	—	—	1 (0.36)
c.4046G>A p.Gly1349Asp **G1349D**	1 (0.47%)	—	—	1 (0.36)
c.4243−1G>T	1 (0.47%)			1 (0.36)
c.4242+1G>A **4374**+**1G>A**	1 (0.47%)			1 (0.36)
Identified total	211 (98.60)	34 (100.00)	32 (100.00)	277 (98.93)
Unidentified	3 (1.40)	—	—	3 (1.07)

Note

BG, Bulgarian, BGTK, Bulgarian Turk, BGRM, Bulgarian Roma CF patients; HGVS, Human Genome Variation Society nomenclature (www.hgvs.org/mutnomen/); Legacy nomenclature according to the Cystic Fibrosis Mutation Database (www.genet.sickkids.on.ca/app); *N*: number of cases; %: percentage rounded up to max. 2 digits after the full stop (thus may not add up exactly to 100%); this table compiles previous reports (Orenti et al., 2018; Makukh et al., 2010; Radivojevic et al., 2004) with this study. The three novel variants are underlined.

a : complex *CFTR* allele.

## 4 DISCUSSION

This study presents a comprehensive overview of the *CFTR* mutation distribution in a representative cohort of 140 unrelated Bulgarian CF patients (i.e., proportionally representing BG, BGTK, and BGRM populations) originating from all regions of the country (Figure 1).

The lack of significant differences in the course of CF between BG and BGTK populations generally reflects their equal access to medical care. In contrast, BGRM CF population despite being clinically diagnosed at an early age is much younger and has worse nutritional status most likely due to their higher infant/childhood mortality. This issue also reflects their generally lower socioeconomic status (Georgiev, Tomova, Grekova, & Kanev, 2001) and observed relatively worse compliance with therapy compared to BG and BGTK CF families (Table 1). Thus, this study provided a basis for a nationwide public health initiative to improve the quality of care, not only in CF, in BGRM.

Using MPS‐based sequencing we have identified 98.30% of all CF‐causing mutations (Table 2; with legacy mutation nomenclature being further used in the Discussion) in combined cohort of 140 cases. In this regard, 15 variants which were not previously reported (Angelicheva et al., 1997; Bobadilla et al., 2002; Savov, 2011; Savov et al., 1995) were identified as well as three complex alleles (in 4 patients) in accordance with previous publications (Savov et al., 1995) (Table 2). We now comply with the diagnostic standards stipulated by recent ECFS Best Practice Guidelines and can confidently implement multitier CFNBS involving DNA testing (Castellani et al., 2018).

The observed differences between the frequencies of different CFTR variants in BG and BGTK populations could not be statistically assessed due to lower number of BGTK cases (Table 2). Although in BGTK patients the c.1040G>C,p.Arg347Pro was the second most common CFTR variant, it is generally less common in TK proper (Bobadilla et al., 2002). Although according to previous publications (Bobadilla et al., 2002; Savov, 2011) all BGRM patients were reported to be c.1521_1523delCTT,p.Phe508del homozygous, we identified two compound heterozygous patients who retrospectively acknowledged BG and BGTK admixture. Three patients where 1 allele remained undetected have classical form of CF with mean sweat chloride concentrations over 60 mM which shows that pathogenic *CFTR* variants may be present in nonexamined *CFTR* introns or that there are other molecular mechanisms involved, but not covered by the utilized assays and/or bioinformatic algorithms (Chen et al., 2008; Lee et al., 2017).

Lower frequency of the predominant c.1521_1523delCTT,p.Phe508del variant reflects its European North‐to‐S. East gradient, whereas marked allelic heterogeneity is in line with previous reports demonstrating its higher rates in S. European populations (Bobadilla et al., 2002) and the high sensitivity/specificity of the applied *CFTR* genotyping approach. The c.3903C>G,p.Asn1303Lys variant which is the second most frequent one in BG is commonly found in adjacent Greek and in TK CF‐populations (Bobadilla et al., 2002; Orenti et al., 2018). The third most prevalent variant, c.1624G>T,p.Gly542*, is typical for populations around the Mediterranean and is rather frequent in neighboring Greece (Kanavakis et al., 2003) and N. Macedonia (Orenti et al., 2018). The fourth most common variant c.2052_2053insA,p.Gln685Thrfs*4 is rather common in W. Ukraine (Ivády et al., 2014) and in E. Hungary (Makukh et al., 2010), but is underrepresented in neighboring CF populations (Bobadilla et al., 2002; Kanavakis et al., 2003; Radivojevic et al., 2004).

In summary, our data provide a strong basis for improvement of DNA diagnostics of CF, foster provision of reproductive choice in preconception‐, preimplantation‐, and/or prenatal DNA testing, facilitate the introduction of multitier CFNBS and eventually will provide patient stratification for the implementation of CFTR modulator therapy (Mitchell, Jones, & Barry, 2018).

## CONFLICT OF INTEREST

The authors have no conflict of interest that could influence the content or processing of this manuscript.
